# Hematological Profile of Hemoglobin C Disease: A Retrospective Study

**DOI:** 10.7759/cureus.80255

**Published:** 2025-03-08

**Authors:** Ismail Regragui, Hassane Mamad, Najoua El mokhtari, Aziz Woumki, Fatima Dahmani, Souad Benkirane, Azlarab Masrar

**Affiliations:** 1 Central Laboratory of Hematology, Ibn Sina Hospital, University Mohamed V of Medicine, Rabat, MAR

**Keywords:** anemia, consanguinity, hemoglobin c, hemoglobinopathies, hplc, target cells

## Abstract

Introduction: Hemoglobinopathies are genetic disorders characterized by qualitative or quantitative abnormalities in globin chain synthesis. This study focuses on Hemoglobin C (HbC) disease, a structural hemoglobinopathy with diverse clinical and hematological manifestations. HbC disease is particularly relevant in populations with high consanguinity rates, where its phenotypic expression and associated complications warrant further investigation.

Objective: The aim of this study is to describe the hematological profile of patients with HbC disease diagnosed at the Central Hematology Laboratory of Ibn Sina University Hospital over a two-year period.

Materials and methods: A retrospective, descriptive study was conducted on 37 cases of HbC disease identified between November 2022 and November 2024. The study population included AC heterozygotes, CC homozygotes, SC compound heterozygotes, and HbC/beta-thalassemia combinations. The hematological evaluation comprised complete blood counts, reticulocyte counts, blood smear analysis, and hemoglobin fraction quantification using high-performance liquid chromatography (HPLC).

Results: The study identified four distinct HbC phenotypes: heterozygous AC (HbAC), homozygous CC (HbCC), compound heterozygous SC (HbSC), and HbC/beta-thalassemia combinations. The SC phenotype was associated with the most severe hematological abnormalities, including significant hemolysis and anemia. Variations in red blood cell morphology and hemoglobin fractions were observed across phenotypes, with elevated fetal hemoglobin (Hb F) levels noted in HbSC patients.

Conclusion: This study highlights the phenotypic diversity of HbC disease in a Moroccan population, emphasizing the role of consanguinity and genetic background in disease expression. The findings underscore the importance of tailored diagnostic and management strategies to address the burden of hemoglobinopathies in high-consanguinity regions.

## Introduction

Hemoglobinopathies are a group of inherited genetic disorders, transmitted in an autosomal recessive pattern, characterized by qualitative and/or quantitative abnormalities in the synthesis of globin chains. Qualitative defects result in the production of structurally abnormal hemoglobin variants, while quantitative defects lead to reduced or absent synthesis of globin chains. Hemoglobin C (HbC) disease, a qualitative hemoglobinopathy, arises from a point mutation in the beta-globin gene, where glutamic acid is replaced by lysine at the sixth position. This autosomal recessive mutation leads to the partial or complete replacement of normal hemoglobin A (HbA) with HbC. Although HbC disease is generally considered a mild condition, it can present with mild to moderate hemolytic anemia, splenomegaly, and an increased risk of infections. The disorder is most prevalent in malaria-endemic regions of West Africa, where heterozygous carriers (HbAC) may have a selective advantage against malaria due to the reduced susceptibility of HbC-containing red blood cells to Plasmodium falciparum infection [1,2].

This study aims to contribute to existing knowledge by describing the hematological profile of 37 individuals diagnosed with HbC disease at the Central Hematology Laboratory of Ibn Sina University Hospital over a two-year period. By analyzing laboratory characteristics, including hemoglobin fractions and erythrocyte indices, we seek to better understand the phenotypic expression of HbC disease in the Moroccan population. Specifically, we aim to explore the distribution of different HbC phenotypes, such as heterozygous AC (HbAC), homozygous CC (HbCC), compound heterozygous SC (HbSC), and HbC/beta-thalassemia (HbC/β-thalassemia) combinations, along with their associated hematological manifestations. This approach focuses on the laboratory findings to provide insights into the diversity of HbC disease and its impact on hematological parameters.

Our findings have critical implications for public health strategies, particularly in regions with high consanguinity rates. By elucidating the interplay between genetic background and HbC disease expression, this work seeks to refine diagnostic protocols, improve genetic counseling, and inform targeted interventions to mitigate the burden of hemoglobinopathies in Morocco and similar populations.

## Materials and methods

This retrospective, descriptive study analyzed 37 cases of HbC disease diagnosed at the Central Hematology Laboratory of Ibn Sina University Hospital over a two-year period, from November 2022 to November 2024. The study included all hemoglobin C profiles, including AC heterozygosity (HbAC), CC homozygosity (HbCC), compound SC heterozygosity (HbSC), and HbC/β-thalassemia combinations.

A comprehensive hematological evaluation was performed for each case, including a complete blood count (CBC) with reticulocyte count, peripheral blood smear examination, and separation and quantification of hemoglobin fractions using high-performance liquid chromatography (HPLC). The HPLC analysis was conducted using the Bio-Rad D-10 analyzer, a widely recognized and reliable system for hemoglobin fraction quantification. 

## Results

The study population comprised 37 patients with HbC disease, with a sex ratio of 1.3 favoring females (21 females versus 16 males). The age at diagnosis ranged widely from 6 months to 67 years, with a median age of 17 years and a mean age of 23.03 ± 20.7 years.

The distribution of HbC phenotypes, as determined by HPLC, revealed four distinct groups: AC heterozygosity (45.9%, n=17), SC compound heterozygosity (43.2%, n=16), CC homozygosity (8.1%, n=3), and HbC/β-thalassemia combination (2.7%, n=1). These findings are summarized in Table 1, which outlines the phenotypic distribution of hemoglobin C disease in the study population.

**Table 1:** Distribution of hemoglobin C phenotypes in the study population (n=37) HbAC: hemoglobin AC; HbCC: hemoglobin CC; HbSC: hemoglobin SC; HbC/β-thalassemia: hemoglobin C/beta-thalassemia

Phénotype	Number of cases	Percentage (%)
HbAC	17	45,9
HbCC	3	8.1
HbSC	16	43.2
HbC/β-thalassémie	1	2.7

In this study, the prevalence and types of anemia among patients with HbC disease were characterized as follows: normochromic microcytic anemia was the most common, affecting 43.2% of patients, followed by normochromic normocytic anemia in 24.3% of cases and macrocytic anemia in 5.4% of cases. Notably, 27% of patients did not present with anemia at the time of diagnosis.

Among the phenotypic groups, the SC compound heterozygosity phenotype exhibited the most severe hematological abnormalities, including severe microcytic anemia, with a mean hemoglobin (Hb) level of 89.4 ± 23.6 g/L and a mean corpuscular volume (MCV) of 82.4 ± 9.07 fL. This phenotype was followed, in descending order of severity, by CC homozygosity, AC heterozygosity, and HbC/β-thalassemia combinations. 

Reticulocytosis was observed in 75% of SC compound heterozygotes, 66% of CC homozygotes, and 6% of AC heterozygotes. This finding highlights the hemolytic nature of anemia in these individuals.

The hematological abnormalities observed in the study population are summarized in Table 2, which provides a detailed comparison of key parameters across the different phenotypic groups. The table includes mean values ± standard deviation (SD) and 95% confidence intervals (CI) for red blood cell count (RBC), Hb levels, MCV, mean corpuscular hemoglobin concentration (MCHC), and reticulocyte count. 

**Table 2:** Hematological profile of different hemoglobin C variants RBCs: red blood cells; Hb: hemoglobin; MCV: mean corpuscular volume; MCHC: mean corpuscular hemoglobin concentration; HbAC: hemoglobin AC; HbCC: hemoglobin CC; HbSC: hemoglobin SC; HbC/β-thalassemia: hemoglobin C/beta-thalassemia; SD: standard deviation; CI: confidence interval; N/A: not applicable

Parameter	Phenotype	Mean ± SD	95% CI (Lower Bound)	95% CI (Upper Bound)
RBCs (10¹²/L)	HbAC	4.5 ± 0.97	4.03	4.96
	HbCC	4.33 ± 0.05	4.27	4.38
	HbSC	3.18 ± 0.85	2.76	3.59
	HbC/β-thalassemia	5.4	N/A	N/A
Hb (g/L)	HbAC	126 ± 22.9	118.6	134.6
	HbCC	110.3 ± 4.7	104.9	115.6
	HbSC	89.4 ± 23.6	76.8	102.0
	HbC/β-thalassemia	112	N/A	N/A
MCV (fL)	HbAC	79 ± 10.89	73.82	84.17
	HbCC	74.30 ± 2.67	71.27	77.32
	HbSC	82.4 ± 9.07	77.95	86.84
	HbC/β-thalassemia	59.9	N/A	N/A
MCHC (g/L)	HbAC	348.8 ± 13.4	342.4	355.0
	HbCC	343.7 ± 11.3	330.9	356.4
	HbSC	345.8 ± 14.6	338.6	352.9
	HbC/β-thalassemia	347	N/A	N/A
Reticulocytes (10⁹/L)	HbAC	69.91 ± 38.12	51.78	88.03
	HbCC	147.5 ± 13.50	132.24	162.7
	HbSC	237 ± 113.3	181.4	292.5
	HbC/β-thalassemia	100	N/A	N/A

Blood smear analysis revealed various red blood cell morphological abnormalities across the different phenotypic groups. Target cells were predominantly observed in CC homozygotes, while sickle cells were characteristic of SC compound heterozygotes, and non-specific size (anisocytosis) and shape (poikilocytosis) abnormalities in AC heterozygotes.

The mean HbC levels varied significantly across phenotypic groups: 35.91 ± 7.02% in AC heterozygotes, 43.13 ± 9.81% in SC compound heterozygotes, and 95.97 ± 0.63% in CC homozygotes (Table 3). Notably, patients with HbSC exhibited significantly elevated levels of fetal hemoglobin (9.63 ± 7.97%) compared to other hemoglobinopathy profiles, except for HbC/β-thalassemia. 

**Table 3:** Separation results of hemoglobin fractions Values are expressed as percentages (%) Hb A: hemoglobin A; Hb A2: hemoglobin A2; Hb F: fetal hemoglobin; Hb C: hemoglobin C; Hb S: hemoglobin S; HbAC: hemoglobin AC; HbCC: hemoglobin CC; HbSC: hemoglobin SC; HbC/β-thalassemia: hemoglobin C/beta-thalassemia

	HbAC	HbCC	HbSC	HbC/β-thalassemia
Hb A (%)	59.3±4.42	0	3.3±6.16	0
Hb A2 (%)	3.1±0.47	3.5±0.16	3.1±0.67	4.1
Hb F (%)	1.6±3.73	0.2±0.14	9.6±7.97	5.9
Hb C (%)	35.9±7.02	95.6±0.63	43.1±6.16	90
Hb S (%)	0	0	40.93±9.26	0

In our study, five families were represented by multiple individuals, accounting for 12 out of the 37 cases. Notably, three patients from the same family exhibited two distinct phenotypes at diagnosis: AC heterozygosity and SC compound heterozygosity. This observation underscores the significant role of consanguinity in the proliferation of hemoglobinopathies within the Moroccan context.

## Discussion

HbC disease is the most frequently found hemoglobinopathy after sickle cell disease [3]. HbC appears following a point mutation in the first nucleotide of codon 6 in the beta-globin gene located on chromosome 11. This involves a substitution of guanine with adenine, resulting in the replacement of glutamic acid with lysine. The lysine in the polypeptide chain replaces two negative charges with two positive charges. This makes the blood more viscous and reduces red blood cell lifespan [3]. Unlike sickle cell disease, HbC does not cause intravascular polymerization under low oxygen conditions, and vaso-occlusive crises are rare except in cases of compound heterozygosity with HbS (HbSC) [3].

HbC is present in regions where Hemoglobin S (HbS) is also widespread [4]. Epidemiologically, the frequency of the HbC mutation varies significantly across different populations. In North Africa, particularly in Morocco and Algeria, the mutation frequency ranges between 1% and 10%. In West Africa, including countries such as Ghana, Ivory Coast, and Burkina Faso, the frequency is notably higher, ranging from 20% to 50%. The prevalence of the clinical syndrome is estimated to be between 1 in 3,000 and 1 in 6,000 individuals in populations of African origin [4]. In the United States, the prevalence of the HbC heterozygous state is 2.4% (2,400 per 100,000) in the African-American population [4].

Individuals heterozygous for HbC are asymptomatic and are most often discovered during neonatal or prenatal screening or during family studies searching for abnormal hemoglobin. They may present with moderate microcytosis, but there is neither anemia nor splenomegaly. The HbC level is around 40% with a mean of 35.91 ± 7.02% in our study. This level may decrease when associated with another hemoglobinopathy, particularly alpha thalassemia [5].

In the literature, reports of homozygous HbC patients are rare. The diagnosis of this hemoglobinopathy is often delayed, typically occurring after the age of 16, as observed in 13 out of 15 patients in one series. Clinical manifestations commonly include splenomegaly, present in approximately 90% of affected individuals, and hepatomegaly, observed in one-third of cases. The anemia is generally moderate, accompanied by reticulocytosis, indicative of its hemolytic nature. Based on available data, only one case of spontaneous splenic rupture in a homozygous HbC patient has been documented in the literature [1,6,7]. Red blood cells (RBCs) in CC homozygous patients exhibit a higher MCHC compared to normal RBCs, which promotes HbC crystallization and subsequent deformation of RBCs into rod-shaped forms. However, no rod-shaped RBCs were observed in our study, potentially due to the limited number of homozygous cases identified [8].

The co-inheritance of HbC and HbC/β-thalassemia results in a clinical presentation similar to homozygous HbC, characterized by microcytic, hypochromic RBCs and a tendency toward pseudo-polycythemia. This phenotype underscores the importance of genetic counseling and screening for concurrent thalassemias in patients with HbC [9].

In our study, patients with HbSC exhibited significantly elevated levels of fetal hemoglobin (HbF) compared to other hemoglobinopathy profiles. This increase in HbF is likely a compensatory response to chronic hemolytic anemia, a hallmark of HbSC. Elevated HbF may confer a protective effect by mitigating the severity of clinical manifestations, particularly vaso-occlusive crises, due to its resistance to hemoglobin polymerization. This finding aligns with previous studies suggesting that higher HbF levels are associated with reduced disease severity in sickle cell disorders, as HbF inhibits the polymerization of HbS, thereby reducing the risk of vaso-occlusion and hemolysis [10].

The co-inheritance of HbS and HbC results in a significant sickle cell syndrome, with a clinical presentation resembling that of homozygous sickle cell disease (HbSS). Patients typically exhibit moderate chronic hemolytic anemia, fewer vaso-occlusive crises, and a reduced risk of acute chest syndrome compared to HbSS individuals. However, patients remain highly susceptible to severe and recurrent complications, including epiphyseal osteonecrosis, proliferative retinopathy (which may lead to vitreous hemorrhage and retinal detachment), recurrent splenic infarctions, an increased risk of stroke, and sensorineural hearing loss. Increased blood viscosity in HbSC patients may also trigger vaso-occlusive complications during pregnancy, necessitating tailored clinical management. Despite these challenges, individuals with HbSC disease have a life expectancy exceeding 65 years [3,10].

Diagnosis of HbSC disease is established through a CBC, which typically reveals moderate regenerative normochromic normocytic anemia, occasionally presenting as hypochromic microcytic anemia, as observed in the present study. Interestingly, the MCV values in some patients were lower than those reported in similar studies, a finding that may be attributed to concurrent iron deficiency in these individuals [11]. Blood smear analysis demonstrates the presence of rare target cells and sickle cells. Hemoglobin separation further confirms the diagnosis, revealing HbS levels ranging from 30.2% to 59.2% and HbC levels between 20.6% and 58.1% in our population. In some patients, HbA is detected at percentages not exceeding 11%, likely indicative of recent blood transfusion.

Erythrocyte cytomorphological abnormalities observed on blood smears are highly indicative, particularly the presence of target cells. The incidental identification of target cells on a blood smear should prompt further diagnostic evaluation, including HbC testing and reticulocyte count [8].

The explicit diagnosis of HbC relies on its detection using agarose gel or cellulose acetate electrophoresis. However, this technique lacks specificity, as it cannot distinguish HbC from other hemoglobin variants such as HbE, HbA2, and HbO-Arab. More advanced methods, such as capillary electrophoresis and HPLC, offer superior resolution for separating hemoglobin fractions into well-differentiated peaks. In our study, we used HPLC, which has the added advantage of accurately quantifying different hemoglobin fractions [12,13].

While this study offers valuable insights into HbC disease in a Moroccan population, several limitations exist. The sample size limited the representation of rare phenotypes like HbCC and HbC/β-thalassemia. As a retrospective, descriptive study, clinical data such as pallor, jaundice, splenomegaly, and follow-up information were unavailable. The absence of peripheral blood smear images and HPLC chromatograms restricted the visual validation of findings. Iron status was not assessed, potentially confounding clinical interpretations. Additionally, HPLC results were not confirmed by electrophoresis, which could have improved reliability. As a single-center study, broader validation in multiethnic populations is needed. Despite these limitations, this work enhances the understanding of HbC disease in high-consanguinity regions and provides a foundation for future research on genetic modifiers and tailored healthcare strategies.

## Conclusions

The identification of cytomorphological abnormalities in red blood cells, such as target cells, anisopoikilocytosis, microcytosis, sickle cells, and rod-shaped cells, serves as a critical diagnostic indicator for hemoglobinopathies. These findings necessitate further investigation using advanced techniques like HPLC and capillary electrophoresis. This study delineates the phenotypic spectrum of HbC disease, identifying distinct profiles, including HbAC, HbCC, HbSC, and HbC/β-thalassemia, each characterized by unique hematological and clinical features. The integration of iron status assessment is essential to differentiate hemoglobinopathies from iron deficiency, ensuring diagnostic accuracy and guiding appropriate therapeutic interventions. Elevated HbF levels observed in HbSC patients suggest potential compensatory mechanisms that warrant further exploration. These findings enhance our understanding of the clinical heterogeneity of HbC disease and highlight the importance of comprehensive screening programs in high-prevalence regions. By identifying at-risk couples and facilitating genetic counseling, such programs can significantly reduce the transmission of severe hemoglobin disorders. Future research should focus on elucidating genetic modifiers, exploring the role of HbF in disease modulation, and developing regionally adapted healthcare strategies. This work contributes to global efforts to mitigate the public health burden of hemoglobinopathies, emphasizing the need for multidisciplinary approaches that integrate clinical, genetic, and epidemiological insights.
